# First autochthonous clinical case of *Hepatozoon silvestris* in a domestic cat in Italy with unusual presentation

**DOI:** 10.1186/s13071-022-05534-x

**Published:** 2022-11-23

**Authors:** Giulia Simonato, Vittoria Franco, Giovanna Salvatore, Simone Manzocchi, Giorgia Dotto, Simone Morelli, Marika Grillini, Laura Cavicchioli, Maria Elena Gelain, Eric Zini

**Affiliations:** 1 Department of Animal Medicine Production and Health, University of Padova, Viale Dell’Università 16, 35020 Legnaro, Padova Italy; 2 AniCura Istituto Veterinario Novara, Strada Provinciale 9, 28060 Granozzo Con Monticello, Novara Italy; 3 IDEXX Laboratories, Strada Provinciale 9, 28060 Granozzo Con Monticello, Novara Italy; 4 Faculty of Veterinary Medicine, University of Teramo, Piano d’Accio, 64100 Teramo, Italy; 5 Department of Comparative Biomedicine and Food Science, University of Padova, Viale Dell’Università 16, 35020 Legnaro, Padova Italy; 6 Clinic for Small Animal Internal Medicine, Vetsuisse Faculty, University of Zurich, Winterthurerstrasse 260, 8057 Zurich, Switzerland

**Keywords:** Domestic cat, Infection, Intestinal nodule, *Hepatozoon silvestris*, Italy

## Abstract

*Hepatozoon* spp. is the causative agent of a vector-borne parasitic disease in many animal species. In felids, *Hepatozoon felis, Hepatozoon canis* and *Hepatozoon silvestris* have been molecularly isolated. Hepatozoonosis usually causes asymptomatic infections in domestic cats, but clinical cases have recently been reported in Europe. We describe the first Italian case of hepatozoonosis in a cat with an unusual presentation. An 11-year-old neutered European shorthair cat was urgently hospitalized for intestinal intussusception. Hematology, biochemistry, FIV-FeLV tests, blood smears and molecular investigation targeting the 18S rRNA gene of *Hepatozoon* spp. were performed on blood samples; in addition, histological and molecular investigations were performed to analyze surgical samples to identify *Hepatozoon* infection. *Hepatozoon* gamonts were detected in granulocytes in the blood smear, and *Hepatozoon* spp. DNA was confirmed by PCR on blood. The intussusception was caused by a sessile endoluminal nodule that was surgically removed. Histologically, many elements referring to parasitic tissue forms were identified in the intestinal cells, and then the specimens were molecularly confirmed to harbor *H. silvestris*. This is the first description of symptomatic hepatozoonosis in a domestic cat in Italy. *Hepatozoon silvestris* has been described in wild felids, which are usually resilient to the infection, whereas the domestic cat seems to be more susceptible. Indeed, *H. silvestris* in cats usually presents tropism for skeletal muscle and myocardium with subsequent clinical manifestations. This is the first description of a domestic cat with *H. silvestris* localized in the intestinal epithelium and associated with intussusception.

**Graphical abstract:**

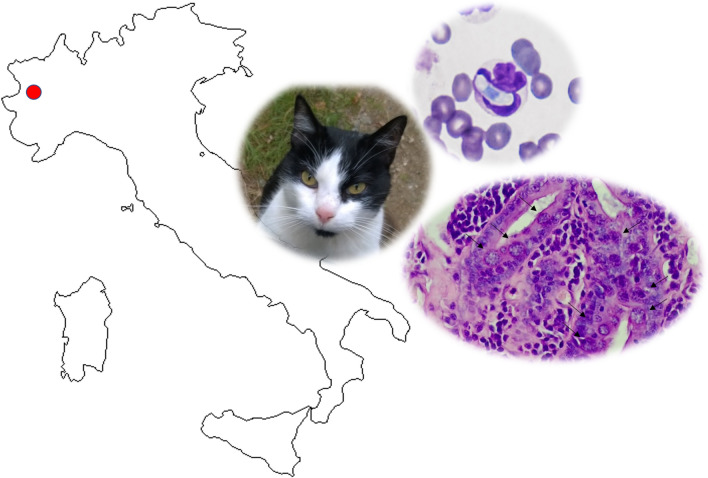

Hepatozoonosis is a vector-borne disease affecting many of animals, including reptiles, birds and mammals; it is caused by an apicomplexan parasite, of which almost 340 species are currently described [1–3]. Almost 50 species are recognized in mammals [1], but comprehensive information regarding their life cycle is known for only a few of them. Usually, the *Hepatozoon* life cycle involves an intermediate and a definitive host represented by a vertebrate animal and an arthropod vector, respectively [4]. In contrast to other vector-borne protozoa (e.g. *Babesia* spp., *Leishmania infantum*) transmitted to humans and animals bitten by infected arthropods, in hepatozoonosis, the vertebrate host becomes infected through the ingestion of infected arthropods [1, 2]. In the vertebrate host, the asexual replication of *Hepatozoon* takes place, generating intracellular gamonts that circulate in the bloodstream. The vectors, mostly represented by ticks, ingest *Hepatozoon* gamonts through blood-feeding from infected animals, and sexual replication takes place within the ticks, ending in the production of mature oocysts that are ready to infect a new vertebrate host and complete the life cycle when the arthropod will be ingested [2]. Interestingly, other transmission routes have been reported, e.g. in the *Hepatozoon canis* and *Hepatozoon felis* life cycles, vertical intrauterine transmission is described, and in the *Hepatozoon americanum* life cycle, predation (i.e. the ingestion of infected prey) has been proven to be an additional transmission route [2, 5, 6].

In felids, hepatozoonosis is still mostly unknown worldwide, but recent epidemiological studies and case reports are raising attention about this parasite and its pathogenicity [7, 8]. Currently, three species of *Hepatozoon* are recognized in wild and domestic felids in Europe, i.e. *Hepatozoon felis*, *Hepatozoon canis* and *Hepatozoon silvestris* [3, 9–11], but no information about the involved arthropod vectors are available, even if ticks seem to be the most likely arthropod vector [12–15]. Usually, hepatozoonosis in domestic and wild felids is considered subclinical, despite replicative forms (i.e. meronts) being found in skeletal muscles and in the myocardium of infected felids [2, 16, 17]. Recently, two cats with clinical signs were described in Central Europe; the first cat was presented with severe and fatal myocarditis and *H. silvestris* infection [7], and the second cat was in poor general condition and exhibited lethargy, anorexia, icterus, a painful abdomen, fever, ruffled hair and *H. felis* infection [8]. This study describes, for the first time in Italy to our knowledge, a clinical and survival case of hepatozoonosis with an unusual presentation in a domestic cat.

An 11-year-old neutered European shorthair cat living in a cat household with > 30 individuals in a pre-alpine area at 700 masl (45°15′98’’N, 7°40′56’’E) and having free outdoor access was examined for its yearly routine veterinary check-up (day -7). A tick was found attached to the cat’s neck and was removed; then, the cat was treated with fipronil. The blood count presented some alterations in red blood cell, reticulocyte and monocyte total counts, and serum albumin was low (IDEXX Laboratories, Italy); no other remarkable findings were observed (Table 1). Five days later, the cat presented mild depression and loss of appetite; in a few days, a worsening of the clinical signs was observed, with anorexia, severe depression and vomiting. The cat was admitted to the hospital (day 0), and no particular findings were revealed during the physical examination such as fever, abdominal mass or pain. A complete hemato-biochemical profile (IDEXX Laboratories, Italy) and an abdominal ultrasound were performed.

**Table 1:** Hemato-biochemical profiles from the first check-up (1 week before the surgery) to the recovery of the *Hepatozoon-*infected cat

	Reference ranges	Days			
		− 7	0	+ 4	+ 13
**Blood count**					
RBC	7.1–11.5 M/µl	6.9	7.0	5.3	5.0
Hct	28.2–52.7%	30.4	28.8	20.9	19.5
Hb	10.3–16.2 g/dl	10.8	10.3	7.9	7.3
MCV	39–56 fl	44.4	41.4	39.3	39.3
MCH	12.6–16.5 pg	15.8	14.8	14.8	14.7
MCHC	28.5–37.8 g/dl	35.5	35.8	37.8	37.4
Reticulocytes (total count)	K/µl	115.1	25	10.1	32.2
WBC	3.9–19 K/µl	15.7	20	15.6	14.2
Neutrophils (total count)	2.62–15.17 K/µl	13.368	14.763	13.706	12.581
Band neutrophils (total count)	0–300/µl	0	2195	0	0
Lymphocytes (total count)	0.85–5.85 K/µl	1.443	1.795	0.749	0.895
Monocytes (total count)	0.04–0.53 K/µl	0.706	1.197	1.124	0.653
Eosinophils (total count)	0.09–2.18 K/µl	0.157	0	0.03	0.07
Basophils (total count)	0–0.1 K/µl	0	0	0	0
PLT	155–641 K/µl	259	266	211	183
Notes			Rouleaux (+++) Burr cells (++) Anisocytosis (+) *Hepatozoon* gamont in neutrophils Platelet aggregates	Rouleaux (+) Heinz bodies (+) Doehle bodies Neutrophils: foamy (+) and basophilic (++) cytoplasm	Rouleaux (+) Platelet aggregates
*Instrument: Sysmex XT2000iV, Sysmex, Kobe, Japan*					
**Biochemical profile**					
Glucose	63–140 mg/dl	85	80	84	97
SDMA	0–14 µg/dl	12	12	19	11
Creatinine	0.9–2.3 mg/dl	1.5	1.6	1.0	0.8
BUN	16–38 mg/dl	23	67	31	18
Phosphates	2.48–6.81 mg/dl	4.34	5.26	4.02	4.02
Calcium	8.82–11.62 mg/dl	8.82	8.42	8.0	8.82
Magnesium	1.46–2.67 mg/dl	2.19	3.4^a^	2.19	1.7
Sodium	147–159 mmol/l	149	140	149	154
Potassium	3.3–5.8 mmol/l	4.8	4.8	5.0	4.5
Chloride	109–129 mmol/l	115	94	116	122
Total protein	5.9–8.7 g/dl	6.2	6.7	5.8	6.8
Albumin	2.7–4.4 g/dl	2.4	2.5	1.9	2.2
Globulins	2.9–5.4 g/dl	3.8	4.2	3.9	4.6
Albumin/globulins	> 0.57	0.64	0.60	0.48	0.49
ALT	27–175 U/l	38	31	51	34
AST	14–71 U/l	29	83	49	26
ALP	12–73 U/l	35	23	60	37
GGT	0–5 U/l	< 1	2	< 1	< 1
Bilirubin (total)	0–0.4 mg/dl	0.2	< 0.1	0.3	0.2
Cholesterol	86–329 mg/dl	123	196	199	149
Triglycerides	21–432 mg/dl	33	60	45	34
Lipase	0.1–45 U/l	nd	21	nd	14
CPK	52–542 U/l	111	2371	1156	313
Urinary creatinine	mg/dl	373	224	nd	nd
Urinary total protein	mg/dl	61.6	42.4	nd	nd
PU/CU	< 0.33	0.2	0.2	nd	nd
Fructosamine	137–286 µmol/l	148	305	150	150
General notes			Vomiting, anorexia, stress, severe depression		Good general conditions
*Instrument: Beckman-Coulter, Brea, CA, USA*					

*RBC* red blood cell count, *Hct* hematocrit, *Hb* hemoglobin, *MCV* mean corpuscular volume, *MCH* mean corpuscular hemoglobin, *MCHC* mean corpuscular hemoglobin concentration, *WBC* white blood cell count, *PLT* platelet count, *SDMA* symmetric dimethylarginine, *BUN* blood urea nitrogen, *ALT* alanine transferase, *AST* aspartate transferase, *ALP* alkaline phosphatase, *GGT* gamma-glutamyl transferase, CPK creatine phosphokinase, *PU/CU* urine protein to creatinine ratio, nd Not done

^a^Hemolytic serum

The blood count showed an inflammatory leukogram with left shift and monocytosis, and the biochemical profile presented several alterations (i.e. electrolytes, CPK, AST, fructosamine) reported in Table 1. Microscopy of May Grunwald Giemsa-stained blood smear revealed single ovoid inclusions in neutrophils attributable to *Hepatozoon* gamonts (11.2 × 5.1 μm, with an ovoid central nucleus, Fig. 1); subsequently, the protozoan parasite was confirmed at the genera level (i.e. *Hepatozoon* spp.) on a blood sample by PCR targeting the 18S-rRNA gene with a cycle threshold (Ct) of 36.3 (IDEXX Laboratories, Germany).

**Fig. 1:**
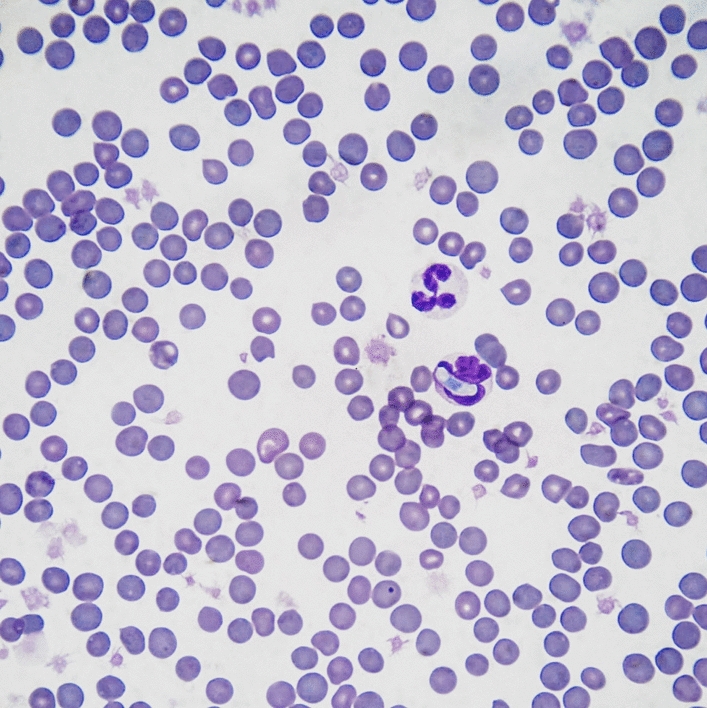
A gamont of *Hepatozoon* sp. in a granulocyte. The blood smear was stained with May-Grünwald Giemsa

Additionally, the cat tested negative for feline immunodeficiency virus (FIV) antibodies and feline leukemia virus (FeLV) antigens (SNAP FIV-FeLV Combo test, IDEXX Laboratories, Inc.). The results were also confirmed by molecular investigations targeting the FeLV proviral DNA (IDEXX Laboratories, Germany). The abdominal ultrasound identified an intestinal intussusception; thus, the cat was urgently admitted to the operating room for a laparotomy.

At surgery, a jejunal endoluminal pedunculated nodule of approximately 1.5 cm, almost occluding the intestinal lumen and causing intussusception, was found. The biochemical parameters improved and normalized within 13 days after surgery (Table 1). The cat was administered a 30-day regimen of oral doxycycline at 5 mg/kg bid. Blood PCR was performed 10 days after the end of doxycycline administration (day + 40), and the result was positive (Ct = 36.9) for the targeted *Hepatozoon* DNA (IDEXX Laboratories, Germany); 1 month later (day + 70), it finally became negative. The time line of the events and their brief descriptions are reported in Fig. 2.

**Fig. 2:**
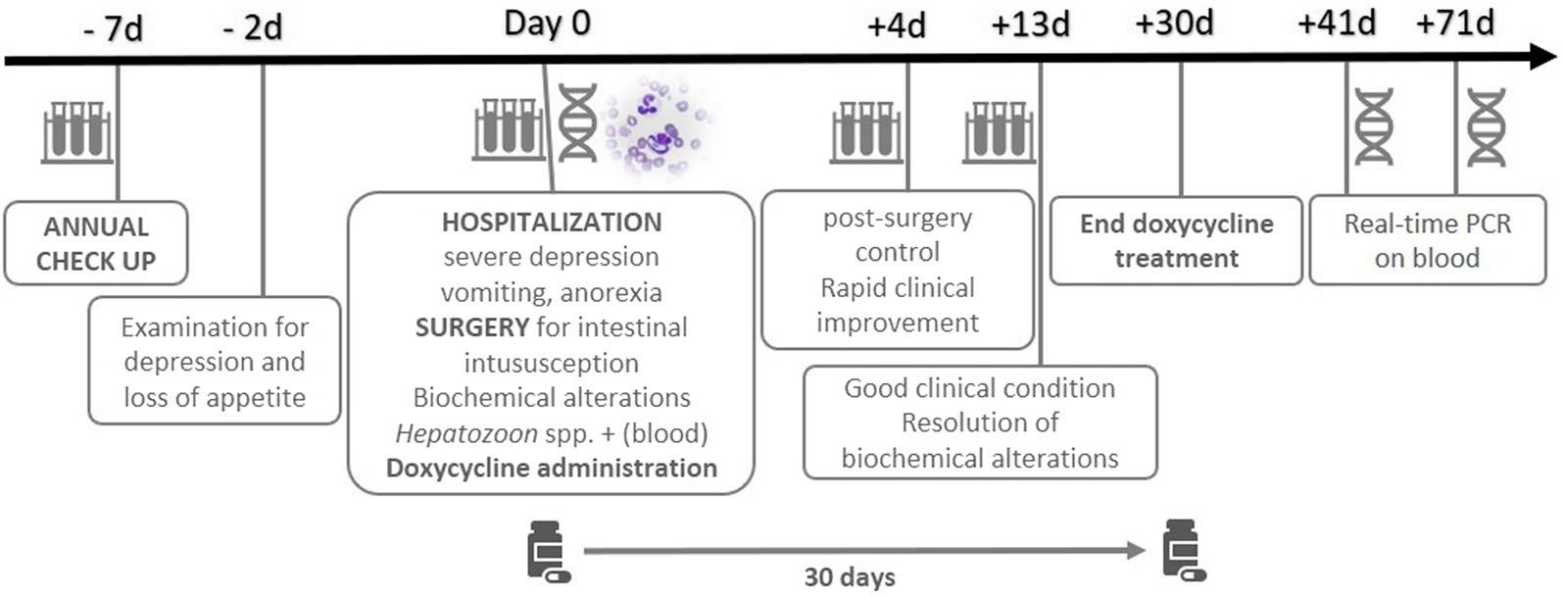
Descriptive time line of clinical conditions, laboratory analyses and treatment of *Hepatozoon*-infected cat

No other vector-borne pathogens were tested because the hemato-biochemical profile and the rapid worsening of clinical conditions were suggestive of acute and severe disease; it was decided to wait for the clinical recovery of the cat before testing for other pathogens; then, since the cat recovered quickly, no other investigations for VBDs were done.

The intestinal nodule was submitted to histopathological and molecular investigations (University of Padova). Tissue sections revealed a severe inflammatory reaction characterized by chronic ulcerative enteritis with polypoid proliferation and severe lymphangiectasia. Many protozoal inclusions were revealed within the enterocytes of the intestinal villi and near the lumen (Fig. 3). The protozoa were roundish, of variable size (with an average size of 15 to 25 µm) and characterized by dark basophilic-staining small nuclei. These forms were referred to as parasitic inclusions of *Hepatozoon* spp. Away from the nodule, along the surgical section in the healthy intestinal tissue, no protozoal inclusions in the enterocytes were observed. Subsequently, to identify the protozoa affecting the intestinal tissue and causing the local host reaction and the nodule, conventional PCR was performed, targeting the 18S-rRNA of *Hepatozoon* spp. with primers described by Tabar et al. [18]. A positive (sequenced DNA of naturally infected cat) and negative (no DNA added) control was added to each PCR. The PCR products were sequenced (Macrogen, Spain) in both directions with the same primers used in PCR. The sequences were compared with those already deposited in GenBank by BLAST software (https://blast.ncbi.nlm.nih.gov/Blast.cgi). Sequence analysis revealed the presence of *Hepatozoon silvestris* (100% homology, accession number: KY649445.1).

**Fig. 3:**
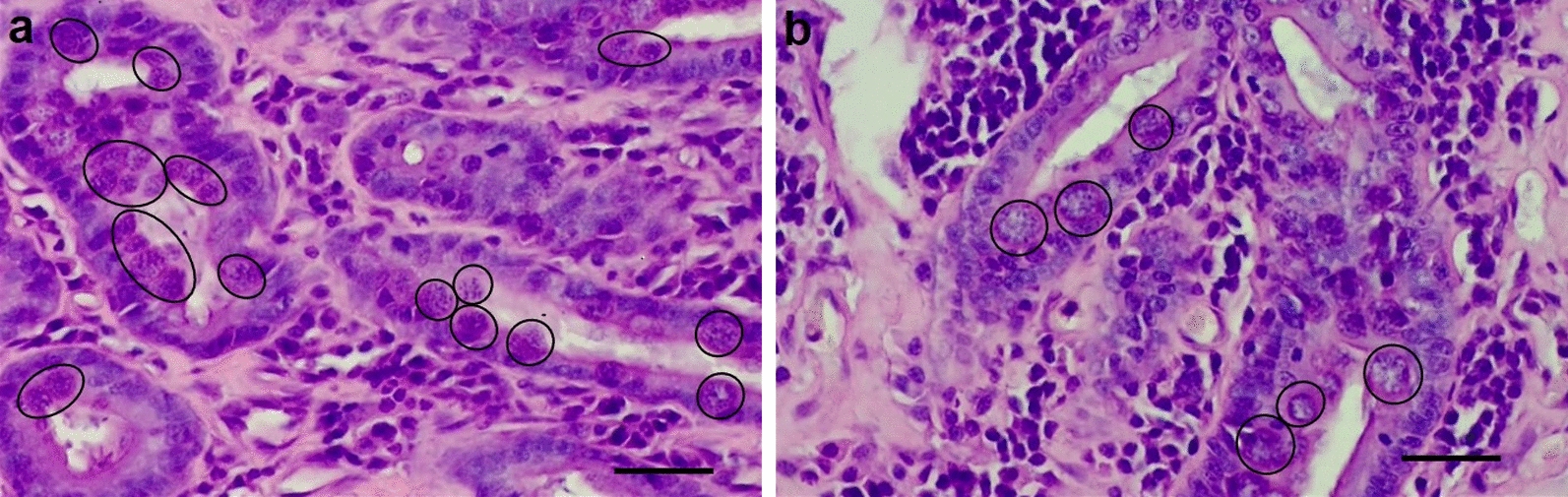
**a**–**b** Histological sections of cat intestinal nodule: protozoan inclusions in the enterocytes (black circles). Hematoxylin-eosin staining. Bar 40 µm

Epidemiological studies reported the detection of *H. silvestris* DNA in wildcats from Bosnia and Herzegovina [3, 21] and in domestic cats from northeastern [10], southern Italy [9] and central Europe [7, 11]. This study describes the case of a cat coming from a hilly area in northwestern Italy close to the Swiss borders, where a fatal case of myocarditis caused by *H. silvestris* in a cat was recently reported [7] and where the presence of wild felids such as the European lynx is frequently described [22, 23]. European lynx and wildcats are already reported as potential reservoirs of several parasites for domestic cat populations sharing the same context of living [10, 21, 24]. Even though in the northwestern Italian regions the presence of lynx and wildcats is rarely observed, the possibility that these animals cross the Alps from highly endemic territories such as neighboring France and Switzerland is probable, as already demonstrated in recent years [23]. The pre-alpine area where the case report took place is close to the French border, and the outdoor lifestyle of the cat suggests exposure to the risk of sharing parasites and arthropod vectors with the sylvatic environment.

Recent studies reported the identification of *H. felis* DNA in *Rhipicephalus sanguineus* sensu lato and *Rhipicephalus turanicus* ticks [12, 14, 25] and *H. silvestris* DNA in *Ixodes ricinus* ticks [15]. This is not sufficient to define the competence role of these ticks as a biological vector. Since *I. ricinus*, already known as the forest tick or castor bean tick, is the most widespread tick in European wild areas, it might be considered to have a potential role in the transmission of *H. silvestris* [7, 13, 26]. The presence of the tick on the cat’s neck during the yearly routine examination (day -7) suggests that exposure to arthropod activity and the ingestion of infected ticks by the cat during fur grooming are possible. Unfortunately, the tick was removed and not conserved; thus, morphological identification and molecular investigations for detecting *Hepatozoon* DNA were not possible; in addition, no other ticks were found after the cat was treated with fipronil. The predation of infected prey, e.g. mostly rodents, was considered another possible route of *Hepatozoon* transmission [2, 5].

In domestic and wild cats, *Hepatozoon* causes a generally subclinical inflammation of skeletal muscles and myocardium [2, 19, 20] as well as elevated values of CPK enzyme in the majority of affected subjects [2, 16, 17]. This finding was observed in our case study, suggesting a potential involvement of skeletal muscles. In cats, the level of parasitemia is generally low and not correlated with the infection burden and the presence of meronts in muscle tissues, and the reason is not yet clear [16, 19]. Neutrophils containing gamonts are usually < 1% [2, 16], as observed in our blood smear evaluation. Specifically, rare gamonts were identified only in the blood smear performed during hospitalization; in all subsequent blood smears, not one gamont was observed.

The intestinal intussusception was generated by a sessile endoluminal nodule, which could have been due to (1) the inflammatory local response to the parasite's penetration through the intestinal mucosa and/or (2) an inflammatory process that was already present where the *Hepatozoon* found a good substrate for replicating. Considering the *H. canis* life cycle already described by Baneth et al. [4], the parasitic inclusions (15–25 µm) found in the histological sections of the sessile nodule, even if smaller than those reported in the literature, could be referred to as protozoan replicative forms such as meronts of *H. silvestris*, suggesting that the nodule was probably the first site of protozoan replication. In addition, the altered values of CPK and AST at day 0 suggested light skeletal muscle damage (i.e. subclinical myositis), as previously reported in cats with hepatozoonosis [16, 17], even if, in this particular case, inflammation of the intestinal muscle layer could be hypothesized. At day 0 circulating sodium and chloride concentrations were low probably because of vomiting. In addition, fructosamine concentration was increased and normalized a few days later; although unproven, it is possible that longstanding stress of the disease temporarily and mildly increased glucose levels. The improvement of CPK immediately after surgery supports the hypothesis that the nodule was the source of the clinical signs. Finally, doxycycline therapy seemed to be helpful for the complete recovery of the cat. Other treatment protocols are reported in cats in the literature such as the combination of doxycycline with primaquine, oxytetracycline with primaquine and imidocarb dipropionate with doxycycline [8, 17]. Actually, there were no controlled studies on the treatment of feline hepatozoonosis, and all information is anecdotal with debatable results. The choice to adopt only doxycycline was due to the difficulties of (i) the off-label use of imidocarb dipropionate in Italy and (ii) finding primaquine on the market easily and/or quickly.

Unfortunately, further histological investigations from other muscle sections and organs before and after treatment would have been useful to evaluate the skeletal muscle involvement, infection burden and efficacy of the treatment protocol but would have been unethical. In addition, the cat was treated to ensure its complete recovery without considering the novelty of *Hepatozoon* infection and the scientific publication.

Further investigations are needed to improve the scientific knowledge on *Hepatozoon* infections in felids, particularly in domestic cats, to prevent severe and potentially fatal clinical cases. Increased knowledge regarding the *Hepatozoon* life cycle in wild and domestic felids as long as arthropod vectors are involved would surely be useful for the adoption of adequate preventative measures in cats.

In conclusion, contrary to the other European case [7] in which *H. silvestris* caused fatal myocarditis in a domestic cat, in this report, the patient recovered completely after surgical removal of the “parasitic” nodule and monthly doxycycline therapy. The intestinal intussusception caused the sudden worsening of the clinical conditions, and surgical resolution was necessary to save the cat. However, the intestinal nodule was probably the result of a local inflammatory reaction to limit the *Hepatozoon* penetration, and it became the first site of protozoan replication; its surgical removal helped the cat to rapidly recover. Despite the unusual clinical presentation of this case, surgery should not to be the treatment of choice in every hepatozoonotic infections with intestinal signs and/or ultrasonographic abnormalities. Feline hepatozoonosis is an emerging vector-borne disease, and considering the recent reports of symptomatic cases, monitoring in cat populations is strongly advised.

## Data Availability

All data analyzed during this study are included in this published article.

## Abbreviations

CPK: Creatine phosphokinase
AST: Aspartate transferase
Ct: Cycle threshold
FIV: Feline immunodeficiency virus
FeLV: Feline leukemia virus
VBDs: Vector-borne diseases
